# An acetabular-preserving procedure for pelvic giant cell tumor involving partial acetabulum

**DOI:** 10.1186/s12957-017-1269-2

**Published:** 2017-11-13

**Authors:** Cong Xiao, Yong Zhou, Wenli Zhang, Yi Luo, Chongqi Tu

**Affiliations:** 1 Department of Orthopedics, West China Hospital, No. 37 Guoxue Xiang, Chengdu, 610041 Sichuan People’s Republic of China; 2 Department of Orthopedics, The Third Hospital of Mianyang, No. 190 The East Jiannan Road, Mianyang, 621000 Sichuan People’s Republic of China

**Keywords:** Giant cell tumor, Acetabulum, Bone graft, Surgical treatment, Osteotomy

## Abstract

**Background:**

The management of pelvic giant cell tumors (GCTs) involving the acetabulum remains a challenge for surgeons on how to balance the relative benefits of minimizing recurrence and maintaining postoperative hip function. The present study was to present and evaluate the clinical indications, operative technique, and outcomes of pelvic GCTs involving partial acetabulum treated with multiplanar osteotomy and reconstruction of autogenous femoral head bone grafts combined with cementless total hip arthroplasty (THA).

**Methods:**

We retrospectively reviewed seven patients with pelvic GCTs involving partial acetabulum who underwent multiplanar osteotomy and reconstruction of autogenous femoral head bone grafts combined with cementless THA from January 2010 to October 2014. We assess the outcome including the bone graft healing, nonunion, hardware failure, infection, tumor recurrence, and metastasis. And the functional outcome was evaluated by the Musculoskeletal Tumor Society (MSTS)93 score.

**Results:**

All patients were followed up for a mean of 38.1 months (range 26–61 months). All bone grafts are union. No failure of acetabular components, wound healing problem, or deep infection was suspected. No patient experienced metastasis. Recurrence was observed in one out of seven patients, treated by extended resection and implanting iodine ions in the surgical area. The mean MSTS93 score was 29.4 (range 28–30). All patients were disease-free and resumed activities of daily living at the most recent follow-up.

**Conclusions:**

As long as one of the two columns is retained and the resulting defect does not exceed the supra-acetabular line, multiplanar osteotomy and reconstruction of autogenous femoral head bone grafts combined with cementless THA is a viable strategy for the treatment of pelvic GCTs involving partial acetabulum. However, a large-scale prospective clinical study is still needed to verify these procedures.

## Background

Giant cell tumors (GCTs) are benign but locally aggressive tumors with a relatively high rate of recurrence if not appropriately managed. GCTs involving the pelvis are extremely rare, accounting for only 1.5–6% of all GCTs of bones [1, 2]. Treatment modalities include intralesional curettage with or without adjunctive procedures and wide resection [2–5]. The options of curettage preserving the integrity of the pelvis can lead to an excellent functional outcome but a high recurrence rate, whereas wide resection has a low rate of recurrence but increases surgical morbidity with complications [3, 4]. So, the management of pelvic GCTs involving the acetabulum remains a challenge for surgeons on how to balance the relative benefits of minimizing recurrence and maintaining postoperative hip function on account of their infrequency and the anatomic complexity of the pelvis [6]. Owing to the local aggressiveness of GCTs, the initial surgical treatment is of vital importance for recurrence of the tumor in the pelvic region often makes it unresectable [3]. So, even with the high rate of associated complications, wide resection is still recommended by several authors [3, 6, 7].

The high rate of failure following traditional resection to region II of the pelvis is attributed to the extensive bone excised, no ideal implants achieving long-term stable fixation, and vulnerability to infection, etc. [3, 6]. To decrease the risk of complications, some authors attempt to preserve the host bone as much as possible by minimizing the resection of healthy tissue surrounding the tumor. The technique “multiplanar osteotomy with limited margins” has been described by Avedian et al. [8], who used this modality to successfully treat the selected patients with high-grade bone sarcomas by making angled bone cuts around a tumor; this preserves as much host bone as possible with the goal of minimizing bone and soft tissue ablation. Similarly, the technique of multiplanar osteotomy was successfully performed by Lam et al. [9] and Gerbers and Jutte [10] for periacetabular neoplasm in selected patients. However, there was no reconstruction for the resulting defect following resection of periacetabular bone; this may contribute to some hip instability, accelerated osteoarthritis, and postoperative hip dislocation [9, 10]. Autogenous femoral head bone grafts have been widely applied for reconstruction of acetabular deficiency in cementless total hip arthroplasty (THA) for developmental dysplasia of the hip [11, 12], and the long-term results are satisfactory. To our knowledge, there has been no such research regarding use of multiplanar osteotomy and reconstruction of autogenous femoral head bone grafts combined with cementless THA for pelvic GCTs involving partial acetabulum.

We performed the above technique in selected patients with pelvic GCTs involving partial acetabulum. The present study was to review the outcome of local recurrence, function, and any associated complications.

## Methods

### Patients

Between January 2010 and October 2014, 26 patients with histologically GCTs of the pelvis were treated with surgery at our orthopedic oncology institution. In the present study, we included patients with the following criteria: (1) tumors were located in region III-II (ischiopubic region with acetabular extension) according to the classification of pelvic tumors of Enneking and Dunham [13]; (2) tumors not extending proximally beyond the supra-acetabular line; (3) no prior management of the tumor; (4) definitive pathological diagnosis of GCT; (5) complete data consisted of clinical notes, radiographic imaging, and pathologic reports; and (6) minimum follow-up of 24 months after surgery. Of the 26 patients, seven met the indication and underwent wide resection in region III, multiplanar osteotomy resection with limited wide margins in region II, and autogenous femoral head graft reconstruction for the residual defect of acetabulum in conjunction with cementless THA. In this series, there were six males and one female; the average age of the patients at presentation was 39.7 years (range 35–44 years). The current study included region III (ischium) + II (partial acetabulum) in six patients, and region III (pubis) + II (partial acetabulum) in one patient. According to the radiographic system of Campanacci et al. [1], one patient was a grade II lesion and six were grade III lesions (Table 1).

**Table 1:** Patient demographics and results

Case	Age (years)/gender	Location	Grade	Surgical time (minutes)	Blood losses (mL)	Follow-up (months)	Complication	Recurrence or metastasis	Function (MSTS93)
1	35/M	IA	III	275	1200	61	None	None	28
2	42/M	PA	II	150	600	35	None	None	30
3	38/M	IA	III	300	2500	41	None	Local recurrence	29
4	44/M	IA	III	210	1500	38	None	None	30
5	40/F	IA	III	250	1300	32	None	None	29
6	40/M	IA	III	180	950	26	None	None	30
7	39/M	IA	III	200	1000	34	None	None	30

*M* male, *F* female, *IA* ischium + acetabulum, *PA* pubis + acetabulum

All patients were retrospectively evaluated clinically and radiographically. The follow-ups were performed at regular intervals: 1, 2, 3, and 6 months after surgery, every 6 months until 2 years after surgery, and then every 12 months thereafter. Physical examination, plain radiographs, chest computed tomography (CT), or magnetic resonance imaging (MRI) were obtained at each visit to assess the outcome including bone graft healing, nonunion, hardware failure, infection, tumor recurrence, and metastasis. Functional outcome was evaluated by the Musculoskeletal Tumor Society (MSTS)93 score [14], which measures the pain, function, emotional acceptance, supports, walking ability, and gait. Each of these six parameters was scored from 0 to 5, giving a maximum score of 30.

### Surgical technique

**Fig. 1:**
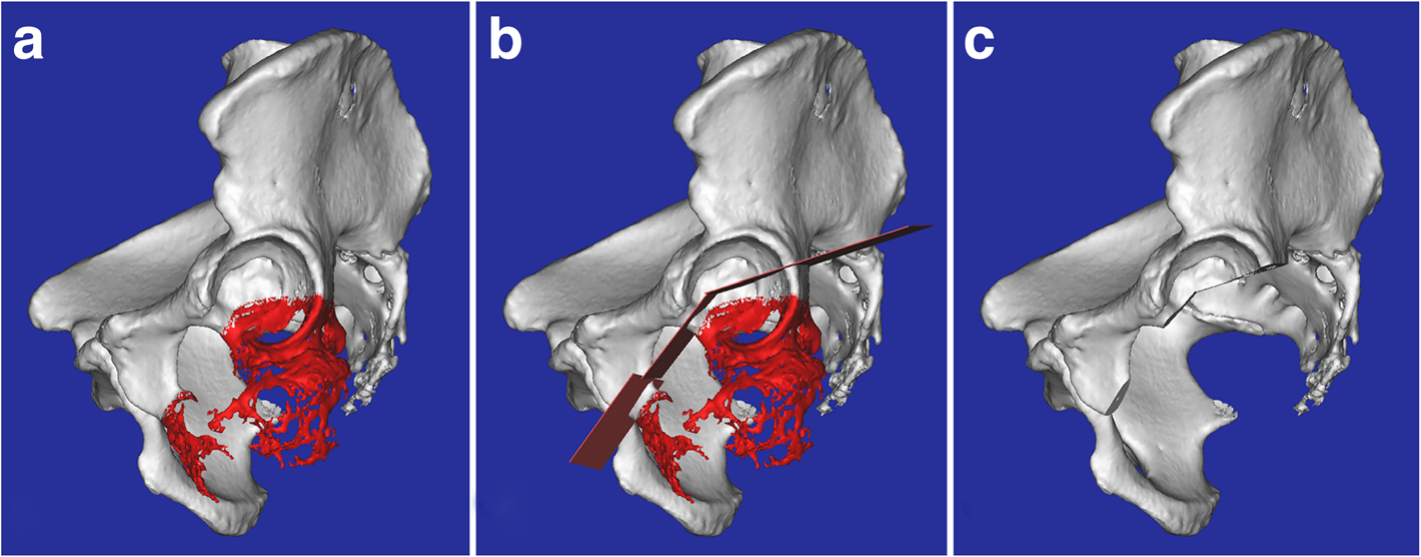
**a** The 3D model of the pelvic bone shows the relationship between the tumor and the acetabulum. The area in red represents the tumor. **b** Multiplanar osteotomy was stimulated with the created plane to determine minimum normal bone and the absence of tumor tissue. **c** The resulting acetabular defect following resection to the tumor is shown

The preoperative planning of the resection of the lesion involving acetabulum was predetermined with the software Mimics version 17.0 (Materialise, Leuven, Belgium). The Digital Imaging and Communications in Medicine (DICOM) files of the patient’s CT scan of the pelvic bone were imported to the software, the tumor image was outlined and segmented, and then the virtual three-dimensional (3D) image of the pelvic bone and the tumor could be created (Fig. 1a). In the window of the 3D view, we can clearly observe the relationship between the tumor and the acetabulum. Multiplanar osteotomy was stimulated with two different created planes to determine minimum normal bone and the absence of tumor tissue (Fig. 1b), as well as > 50% preserved host bone [15].

**Fig. 2:**
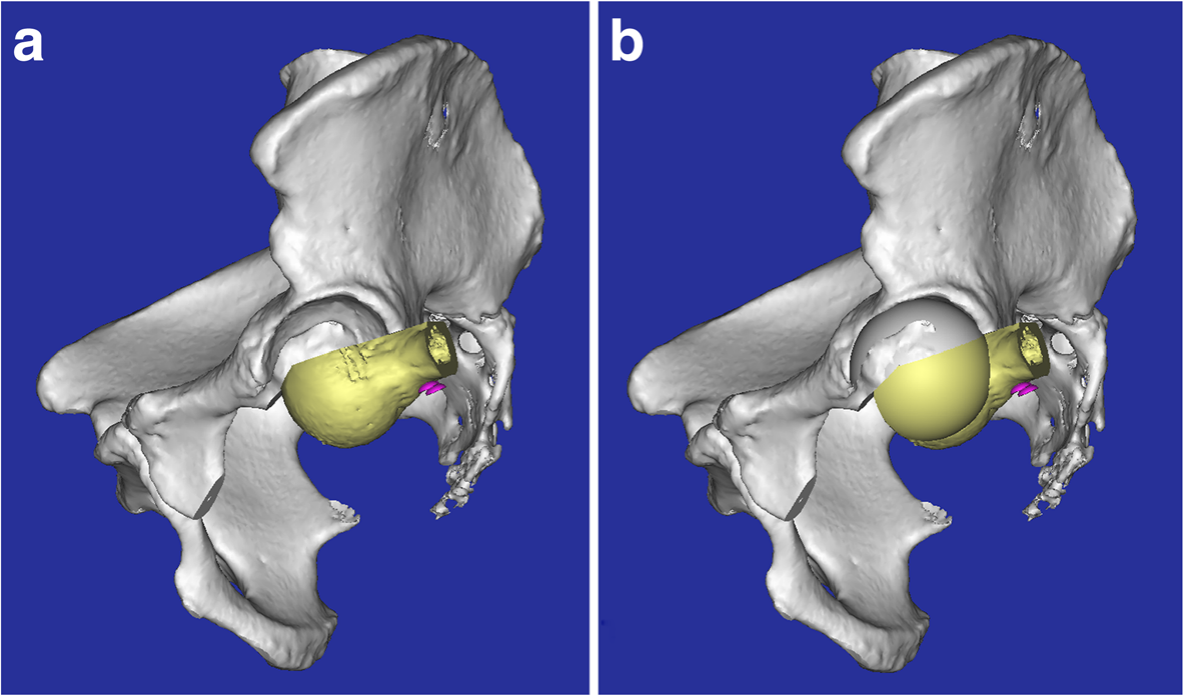
**a** The 3D model shows the femoral head was trimmed to be congruent with the residual host bone of the acetabulum, and then the graft was fixed securely to the host bone with two screws. **b** The reamed socket was prepared

All surgeries were performed by the same team. Under general anesthesia, the patients were placed in the contralateral decubitus position. The posterolateral approach was used for six patients with the tumor located in the ischium and posterior acetabulum, while the anterior approach was applied for one patient with the tumor located in the pubis and anterior acetabulum. Femoral neck osteotomy was carried out in usual fashion, and the femoral head was well preserved. An oscillating saw was used to perform the resection of region III, obtaining a wide margin. With respect to the acetabular osteotomy, a reciprocating bone saw (Stryker®, USA) was used for a multiplanar osteotomy to accurately obtain a limited wide margin according to the preoperative planning (Fig. 1c). Then, the resulting defect following resection of the acetabular lesion was reconstructed with the bulk autogenous femoral head bone graft. The cancellous portion of the femoral head was trimmed with the reciprocating bone saw to be congruent with the residual host bone of the acetabulum, and then the graft was fixed securely to the host bone with two or three partially threaded cancellous screws (Fig. 2a). The socket was prepared and reamed carefully in the usual way (Fig. 2b). An appropriately sized uncemented acetabular component was impacted into place, supplemented with screws. A hydroxyapatite-coated uncemented femoral stem was applied.

### Postoperative management

The patients were allowed to non-weight-bearing stand and walk with two crutches 2 days after surgery. Range of motion exercise of the hip was performed from postoperative week 2. Partial weight-bearing with crutches was encouraged from 4 weeks postoperatively, followed by gradual full weight-bearing.

## Results

**Fig. 3:**
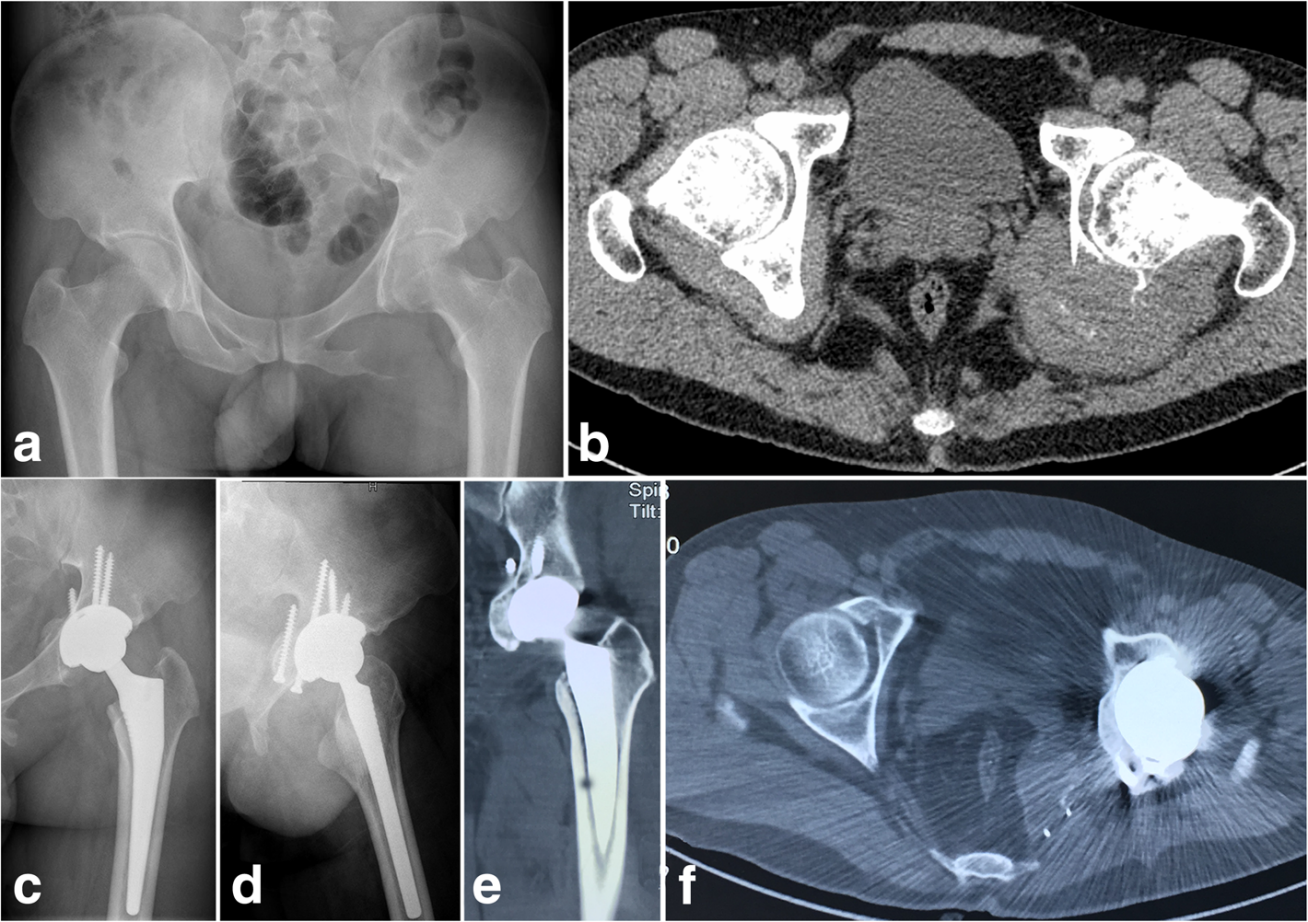
**a** X-ray and **b** axial CT scans of a 44-year-old male show the tumor was located in region III (ischium) + II (posterior acetabulum). **c** Anteroposterior and **d** lateral X-ray at the last follow-up showing no bone graft collapse, absorption, or screw failure. **e** Coronal and **f** axial CT scan at the last follow-up showing bone graft union with the host bone

**Fig. 4:**
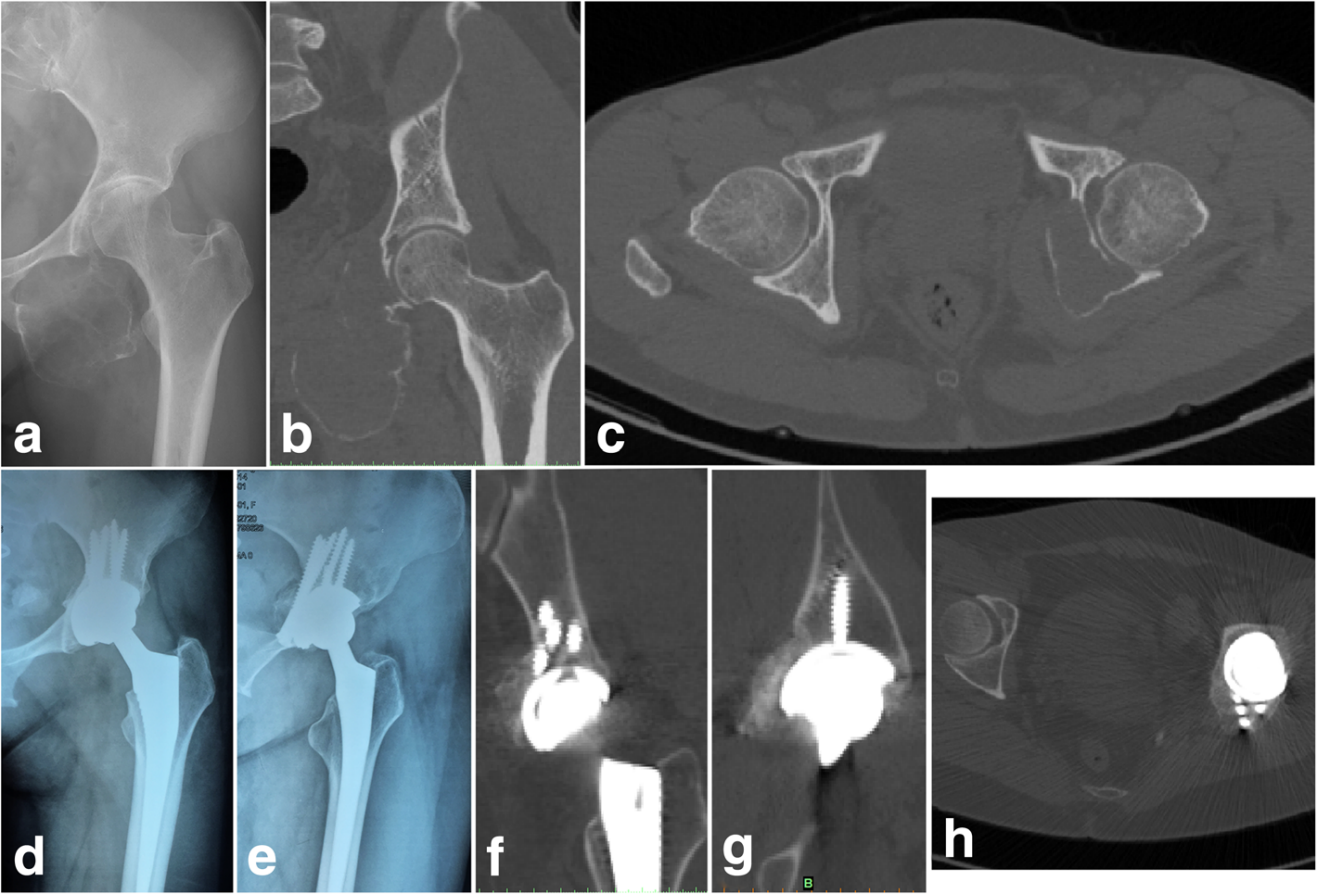
**a** X-ray, **b** coronal, and **c** axial CT scans of a 40-year-old female shows the tumor was located in region III (ischium) + II (posterior acetabulum). **d** Anteroposterior and **e** iliac bone oblique X-ray at the last follow-up showing no bone graft collapse, absorption, or screw failure. **f** Coronal, **g** sagittal, and **h** axial CT scans at the last follow-up showing bone graft union with the host bone

All patients were followed up for a mean of 38.1 months (range 26–61 months). All bone grafts obtained union with the host bone without additional complications (such as nonunion, collapse, absorption, or screws failure). There were no failures of acetabular components. All patients can walk without any aids 6 months after surgery. There was no wound-healing problem. No deep infection was suspected. No patient experienced metastasis. One patient complained of a dull pain in the inguinal region at 15 months postoperatively. Hardware failure and deep infection was excluded by radiographs and hematological examination. Further magnetic resonance imaging confirmed local soft tissue mass. Recurrence was suspected and confirmed by open biopsy. Extended resection of the soft tissue mass was performed, and iodine ions were implanted in the surgical area. At the last follow-up, the mean MSTS93 function score was 29.4 (range 28–30). All patients were pain-free and resumed activities of daily living. Typical cases (cases 4 and 5) are shown in Figs. 3 and 4.

## Discussion

In this study, a series of seven patients with pelvic GCTs involving ischium/pubis and partial acetabulum were carefully screened and treated by multiplanar osteotomy with a limited wide margin and reconstruction of autogenous femoral head bone grafts combined with cementless THA, with the goal of minimizing ablation of healthy bone and enhancing the hip function.

The treatment of pelvic GCTs involving acetabulum is usually difficult and controversial. Intralesional curettage can preserve the integrity of the pelvis and obtain good hip function. However, a systematic review reported that those who had undergone intralesional surgery had a higher rate of local recurrence (33.3%) than those treated by wide resection (2%) [6]. Traditional options of wide resection are to remove the whole bone of region II, which destroy the integrity of the pelvis. A variety of complications such as wound infection, nonunion, bone absorption, deep infection, or hardware failure are obvious; this leads to poor hip function. Until now, few publications have specifically addressed pelvic GCTs involving the region III with partial acetabulum extension [2, 4, 5, 7, 16–20] (Table 2). It remains a challenge to musculoskeletal oncologists on how to balance minimizing the recurrence rates and maximizing the hip functional outcomes.

**Table 2:** Demography of patients with pelvic GCTs involving region III with partial acetabular extension in various studies

Study	Location (number)	Treatment (number)	Complication (number)	Recurrence	Metastasis
Nishida et al. [16]	IA (1)	ILC + phenol + allograft	Migration	None	None
Sanjay et al. [2]	IA (3); PA (1)	ILC + autograft	Infection (2)	3	2
Matsumoto et al. [17]	IA (1)	ILC + cementation	None	None	None
Marcove et al. [18]	IPA (1); IA (1)	ILC + cryosurgery + prosthesis (1); ILC + cryosurgery + radiation + cementation (1)	Sciatic nerve palsy (1)	None	None
Balke et al. [5]	IA (2); PA (2); IPA (2)	ILC + cementation (3); ILC + cryosurgery + bone graft + radiation (1); ILC + hip transposition (1); sole radiation (1)	Screw dislocation (2); femoral head necrosis (1); subluxation of femoral head (1)	None	None
Leggon et al. [4]	IA (3)	WR + iliofemoral arthrodesis	None	None	None
Oda et al. [19]	PA (1)	WR + iliofemoral arthrodesis	None	None	None
Osaka and Toriyama [7]	IPA (1); IA (1)	WR + iliofemoral arthrodesis (1); WR + THA (1)	Opening of ilum (1); infection (1)	None	None
Mnaymneh and Mnaymneh [20]	IPA (1)	Wide amputation	None	None	None
Current study	IA (6); PA (1)	WR + autograft + THA	None	1	None

*IPA* ischium + pubis + acetabulum, *ILC* intralesional curettage, *WR* wide resection

In order to reduce the local recurrence rate and improve the hip function, wide resection should be performed; meanwhile, traditional wide resection of region II should be avoided. In a study of five patients with malignant neoplasms of the periacetabular region, Lam et al. [9] performed acetabular-preserving resections that preserved the weight-bearing acetabulum, and satisfactory outcomes were obtained with a median follow-up of 37 months. Gerbers and Jutte [10] reported one patient with chondrosarcoma of the region III treated by partly resecting the frontal part of the acetabulum with computer assistance to obtain a safe margin and achieved an excellent postoperative function with a follow-up of 3.5 years. In the present study, cases were carefully screened to determine whether the patients were candidate for this type of surgery. With the assistance of computer simulation, we make precise preoperative planning carefully. The planes of osteotomy around the acetabulum were created to make sure a safe margin was achieved when excising the partial acetabulum. At the same time, enough host bone can be preserved for reconstruction. It is of particular importance that the dome of the acetabulum should be retained after osteotomy. Generally, tumors extending proximally beyond the supra-acetabular line are excluded in our study. This method can avoid complex reconstruction, and leads to better hip function than the standard resection of region II. In our series, limited and safe margins are achieved by multiplanar osteotomy with the help of precise preoperative simulation. How to achieve a limited and safe margin should be considered. Computer-assisted surgery, a trustworthy means of navigation, has been reported in resections of pelvic tumors [10]. This can provide precise imaging and achieve desired safe margins. Lam et al. [9] reported that the precise planning of the resection was carried out with computer navigation software. In the current study, we use the Mimics software to visualize and segment the CT images and render 3D pelvic bone. The tumor 3D models can be extracted. Then, the relationship between the tumor and the healthy bone can be obviously displayed. Accurate planes of osteotomy are created in the window of the 3D view. Taking the apex of acetabulum and acetabular fossa as reference during operation, the senior surgeon can easily achieve limited and safe margins with the guide of preoperative simulation. Postoperative biopsy further confirmed a clear margin.

During preoperative planning, it is of vital importance to consider the residual defect following resection and the material that will be utilized to fill this defect. Bulk autogenous graft from the femoral head has been widely used to fill the deficient acetabula in patients with developmental dysplasia of the hip. In patients with the use of cemented acetabular components, the failure rate ranges from 38 to 46% over at least a 10-year follow-up [21, 22], mostly because of asymptomatic loosening or graft collapse. With the advent of cementless acetabular components, the 10-year survival rate without acetabular revision for any reason ranges from 94 to 100% [11, 12, 23]. Certainly, one must be aware that the size of the defect that is filled by the graft may affect survivorship. Several authors recommended that the coverage of the socket by the graft not exceed ranging from 30 to 50% [11, 12, 24]. In this series, only the patients with > 50% preserved host bone following resection of the periacetabular region are included. At the last follow-up, the grafts are union and no acetabular components needed to be revised. The character of our patients differs from that of other reports. So, we cannot make any comparison. Our clinical experiences indicate that as long as one of the two columns is retained and the resulting defect does not exceed the supra-acetabular line, this is a viable method of reconstruction for patients with pelvic GCTs involving partial acetabulum.

Several factors can influence the successful incorporation of the autogenous femoral head bone grafts: First, graft orientation in relation to the host bone is of significance. We always make the portion of the femoral neck in contact with the proximal deficient acetabulum, and the femoral head faces the acetabular fossa. The cortex of the femoral neck can bear greater pressure and provide better holding force when implanting the screws, while the cancellous bone of the femoral head can be easily trimmed and reamed when preparing the socket. Second, it may be technically demanding to match the defect. According to the preoperative simulation, we had a preliminary understanding on how to trim the bone graft. Then, the reciprocating bone saw was well applied to carefully trim the graft to obtain the satisfactory matching of the defect. Third, it is of particular importance that the screws cannot protrude into the socket following reaming of the acetabular fossa. And screw orientation should be close or parallel to the conduction force of the acetabulum for the reason that axial compression of the graft can enhance bone graft incorporation with the host bone. Our preoperative simulation to the placement of the screws can be a good solution to this concern.

In this series, six out of seven patients were grade III lesions at presentation. The probable reasons of the high rate are uncharacteristic clinical presentation, easily confused with low back pain, arthritis, muscle strain, or lumbar intervertebral disc herniation, no visible swelling, and misinterpreted radiographs caused by gas-filled intestines. In the present study, local control was achieved in six out of seven patients. Recurrence occurred in one patient, confirmed by further MRI and open biopsy. It should be noted that this is a grade III lesion, and the recurrent tumor located not in the periacetabulum but in the soft tissue. We believe it might not be possible that no safe margins in the periacetabular region were achieved. The possible reason of recurrence may be inadequate soft tissue margin or the presence of satellite lesions that were unable to be seen by the naked eye.

Several limitations of our study should be noted. First, only a small proportion of cases with pelvic GCTs involving ischium/pubis and partial acetabulum were candidates for this type of surgery. It may have led to deviation over the results of local recurrence, bong graft healing, or failure of acetabular components. Second, although we made a precise preoperative planning relying on computer software to determine the plane of osteotomy, it might include a somewhat subjective process such as the surgeon’s experience with osteotomy. It may be more accurate with the assistance of the osteotomy guide in the future. Third, the follow-up is short; additional local recurrences may be detected with longer follow-up. Nevertheless, 70% of local recurrences occur within 2 years [25]. Fourth, in this retrospective series, no patient was treated with denosumab. This drug is a human monoclonal antibody against the receptor activator of nuclear factor kappa-B ligand, which can shrink the size of large GCTs to facilitate tumor resection [26]. So, it should be strongly recommended that denosumab be subcutaneously administered before surgery, especially in patients with grade III.

## Conclusions

Multiplanar osteotomy and reconstruction of autogenous femoral head bone grafts combined with cementless THA can be performed for patients with pelvic GCTs involving partial acetabulum and not extending proximally beyond the supra-acetabular line. According to computer-aided preoperative simulation, much more host bone was maintained, and the resulting defect was easily reconstructed by autogenous femoral head bone graft. As a result, the grafts are union and no acetabular components needed to be revised. The patient’s postoperative function was similar to that of primary THA. At the same time, the local recurrence rate was reduced. A large-scale prospective clinical study is warranted to verify our results. Nevertheless, the present study describes a viable strategy for treatment of this challenging condition.

## Abbreviations

3D: Three-dimensional
CT: Computed tomography
DICOM: Digital Imaging and Communications in Medicine
F: Female
GCTs: Giant cell tumors
IA: Ischium + acetabulum
ILC: Intralesional curettage
IPA: Ischium + pubis + acetabulum
M: Male
MRI: Magnetic resonance imaging
MSTS: Musculoskeletal Tumor Society
PA: Pubis + acetabulum
THA: Total hip arthroplasty
WR: Wide resection
